# Drive Time Analysis of New Gynecologic Oncology Patients at West Virginia University

**DOI:** 10.13023/jah.0701.08

**Published:** 2025-05-01

**Authors:** Kassandra Whitfield, Ariel Cohen, Krista Pfaendler, Kristy Ward

**Affiliations:** West Virginia University; West Virginia University; West Virginia University; West Virginia University

**Keywords:** Appalachia, gynecologic oncology, rural health, travel time, West Virginia

## Abstract

**Introduction:**

West Virginia is a rural mountain state; access to medical care, especially specialty care, can be difficult for patients.

**Purpose:**

The purpose of this study is to analyze the geographic status and transportation times of new gynecologic oncology patients.

**Methods:**

Home zip codes for new patient gynecologic oncology patients were analyzed for drive times to the cancer center. Zip codes of oncology providers in the state were compared to the location of patients.

**Results:**

A total of 1,097 new gynecologic oncology patients lived within a 240-minute drive of MBRCC; nearly half (48.9%) of them drove greater than 60 minutes. There are large geographical areas of West Virginia without an oncology practice location.

**Implications:**

West Virginians face barriers to gynecologic oncology care secondary to drive times.

## INTRODUCTION

West Virginia (WV) is a rural mountain state; due to this geographical challenge, access to medical care, especially specialty care, can be difficult for patients.^1^ The rural population faces challenges in access to health care including shortages of oncologists and cancer specialists, increased exposure to cancer risk factors, and decreased socioeconomic status as compared to urban residents.^2^–^4^ These factors contribute to the increased incidence and mortality of cancer in this rural populations.^5^

The purpose of this study is to analyze the geographic status and transportation times of new gynecologic oncology patients to the West Virginia University Mary Babb Randolph Cancer Center (MBRCC), located on the campus of the largest tertiary care center in the state.

## METHODS

A list of zip codes of the home address of all new patient visits with the gynecologic oncology providers at the MBRCC from August 2020 – August 2022 was obtained. The zip code list was loaded into the ArcGIS Geographic mapping software online (ESRI, 2023) and a drive time analysis performed. The address of the MBRCC was input as the reference point for the drive time analysis. Travel time areas were calculated with boundaries created at 30, 60, 120, 180, and 240 minutes from MBRCC. The drive time analysis and zip code data points were aggregated to calculate the number of patients who lived within each drive time area.

To evaluate the distribution of oncology providers in WV, we queried the public West Virginia Board of Medicine “Look up a doctor or PA” function.^5^ Physicians with an active license in WV were searched by specialty for gynecologic oncologists, hematologic oncologists, and radiation oncologists. The practice zip codes were recorded for all providers identified with a practice location in WV or the surrounding states (Pennsylvania, Maryland, Virginia, Kentucky, Ohio). The zip codes of the providers located in WV were mapped in ArcGIS along with the zip code locations of the new patients.

## RESULTS

**Figure 1:**
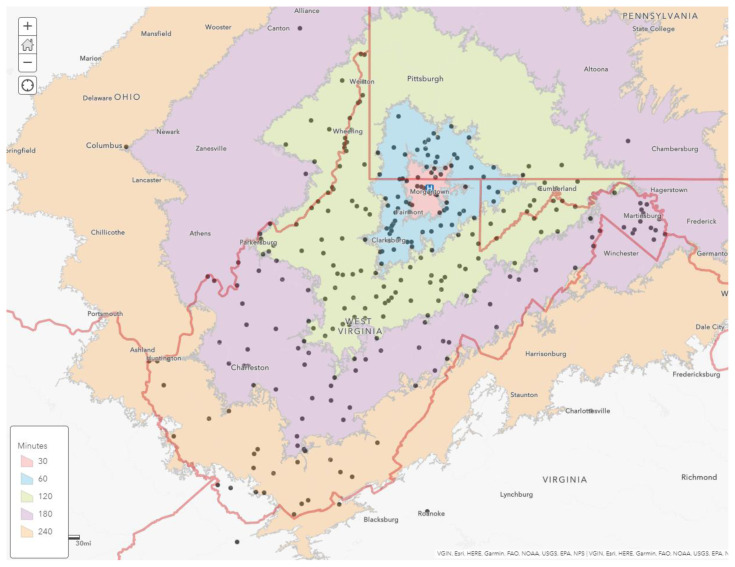
Drive-time Analysis of New MBRCC Patients. NOTE: Each dot represents the home zip code of new patients. Each zip code identified is represented by one dot (each dot may represent more than one patient). The MBRCC is indicated by the blue circle with an “H.”

There was a total of 1,110 new patient visits to the MBRCC gynecologic oncology outpatient clinic from August 1, 2020 – August 31, 2022. A total of 1,097 patients lived within a 240-minute drive of the cancer center **(**Fig. 1). Thirteen patients had addresses that would indicate greater than 240-minute drive time. These patients are not included on the map due to scale. Nearly half (48.3%) of the new gynecologic oncology patients seen at the MBRCC drive greater than 60 minutes to reach the cancer center. Only 22.7% of patients (252) lived within a 30-minute drive of the MBRCC.

**Figure 2:**
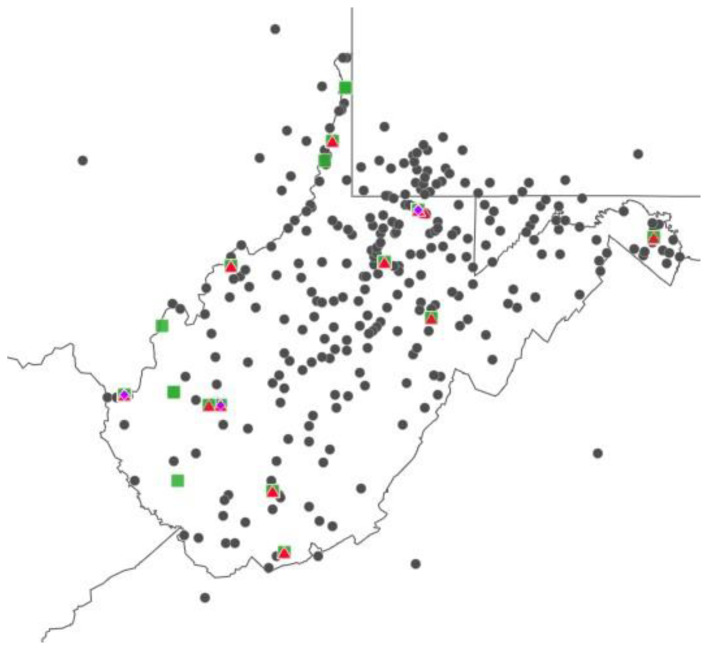
Oncology Providers in West Virginia. NOTE: This is a map of the primary practice location of physicians licensed by the WV Osteopathic Board of Medicine who identified their practice as gynecologic oncology (purple diamond), radiation oncology (red triangle), or as hematologic oncology (green square). Dots represent new patients to the gynecologic oncology practice at WVU MBRCC.

According to the West Virginia Board of Medicine provider search, there were 11 gynecologic oncologists, 105 hematologic oncologists, and 51 radiation oncologists with an active WV license and a primary practice zip code in WV or the surrounding states on the date of query. The map shows the providers within the state of WV **(**Fig. 2). There are 8 licensed gynecologic oncologists at 3 locations, 86 licensed hematologic oncologists at 16 locations, and 38 radiation oncologists at 12 locations within WV. All radiation oncologists are at sites that contain hematologic oncology, and all gynecologic oncologists are at sites containing radiation oncology and hematologic oncology.

## IMPLICATIONS

Most new gynecologic oncology patients travel more than one hour to access care at MBRCC. As WV has a higher incidence of and mortality from gynecologic cancers than the US rate,^6^ this creates significant cost burden to both our patients and the state. Geographic constrictions are not exclusively based on physical distance but can include access to personal or public transportation and sufficient funds for gas. As WV is the only state entirely contained in Appalachia, road conditions are a particular concern when planning surgery and treatment timing. Given the transportation difficulties of these patients, ensuring appropriate follow-up, surgical planning, hospital admissions, chemotherapy and radiation treatments, and surveillance can be hindered and likely contribute to the mortality rate.

Further research should focus on economic impacts of prolonged travel time, infrastructure issues resulting in barriers to care, and treatment delays or termination related to distance. Innovative solutions should be explored including telemedicine, resource distribution, and self or mobile cancer screening methods. This paper should kindle interest in this pursuit.

**SUMMARY BOX**

**What is already known about this topic?**

West Virginia is a rural state and access to gynecologic cancer care can be complicated by the geographical layout of the area and the availability of healthcare resources.

**What is added by this report?**

This report highlight the transportation barriers that patients to a gynecologic oncology clinic face in seeking essential healthcare.

**What are the implications for future research?**

Further research should focus on economic impacts of prolonged travel time, infrastructure issues resulting in barriers to care, and treatment delays or termination related to distance. Innovative solutions should be explored including telemedicine, resource distribution, and self or mobile cancer screening methods.
